# Inhibition of lncRNA NEAT1 induces dysfunction of fibroblast-like synoviocytes in rheumatoid arthritis via miRNA-338-3p-mediated regulation of glutamine metabolism

**DOI:** 10.1186/s13018-022-03295-y

**Published:** 2022-09-01

**Authors:** Mei Zhang, Ning Lu, Hong-Jun Li, Xiao-Yun Guo, Lu Lu, Ying Guo

**Affiliations:** 1 Department of Rheumatology and Immunology, Tianjin Medical University General Hospital, 154 An-Shan Road, Heping, Tianjin, 300052 People’s Republic of China; 2 Department of Breast Medical Oncology, Tianjin Medical University Cancer Institute and Hospital, National Clinical Research Center for Cancer, Key Laboratory of Cancer Prevention and Therapy, Tianjin’s Clinical Research Center for Cancer, Key Laboratory of Breast Cancer Prevention and Therapy, Tianjin Medical University, Ministry of Education, Tianjin, 300060 People’s Republic of China; 3 Department of Rheumatology and Immunology, The Second Hospital of Tianjin Medical University, Tianjin, 300211 People’s Republic of China; 4 Department of Nephrology, The Second Hospital of Tianjin Medical University, Tianjin, 300211 People’s Republic of China; 5 Department of Pharmacy, Tianjin Medical University Cancer Institute and Hospital, National Clinical Research Center for Cancer, Key Laboratory of Cancer Prevention and Therapy, Tianjin’s Clinical Research Center for Cancer, Tianjin, 300060 People’s Republic of China

**Keywords:** Rheumatoid arthritis (RA), Fibroblast-like synoviocytes (FLSs), lncRNA, NEAT1, miR-338-3p, Glutamine metabolism

## Abstract

**Background:**

Rheumatoid arthritis (RA) is a systemic chronic autoimmune disease; cellular glutamine metabolism in fibroblast-like synoviocytes (FLSs) of RA was known to be essential for RA pathogenesis and progression. NEAT1, a long non-coding RNA, functions as an oncogene in diverse cancers. The exact roles and molecular mechanisms of NEAT1 in fibroblast-like synoviocytes (FLSs) of RA patients are unknown.

**Methods:**

Expression of NEAT1 and miR-338-3p was measured by qRT-PCR. lncRNA-miRNA and miRNA-mRNA interactions were predicted from starBase and validated by RNA pull-down and luciferase assay. The glutamine metabolism of FLSs was evaluated by glutamine uptake and glutaminase activity. Cell death in FLSs in response to H_2_O_2_ was assessed by MTT and Annexin V assays.

**Results:**

NEAT1 was significantly upregulated, and miR-338-3p was significantly downregulated in FLSs from RA patients compared to normal FLSs. Silencing of NEAT1 and overexpression of miR-338-3p suppressed glutamine metabolism in FLSs-RA and promoted H_2_O_2_-induced apoptosis. Bioinformatics analysis showed that NEAT1 sponges miR-338-3p to form competing endogenous RNA (ceRNAs), which was verified by RNA pull-down assay and luciferase assay FLSs-RA had an increased rate of glutamine metabolism compared to normal FLSs increased compared to normal FLSs. The results confirmed that GLS (Glutaminase), a key enzyme in glutamine metabolism, is a direct target of miR-338-3p in FLSs-RA. miR-338-3p inhibition of glutamine metabolism was verified by rescue experiments verified. Finally, restoration of miR-338-3p in FLSs-RA expressing NEAT1 overcomes NEAT1-promoted glutamine metabolism and resistance to apoptosis.

**Conclusions:**

This study reveals the essential role and molecular targets of NEAT1-regulated glutamine metabolism and FLSs-RA dysfunction in fibroblast-like synoviocytes of RA and indicates that blocking the molecular pathway via non-coding RNAs may be beneficial for RA patients.

## Background

Rheumatoid arthritis (RA) is known as a chronic autoimmune disease that causes synovial inflammation, joint destruction, and bone erosion [1]. In recent years, early diagnosis of rheumatoid arthritis, the discovery of novel drug targets, and the establishment of therapeutic strategies have improved therapeutic approaches for patients [2]. Currently, treatment strategies for RA include conventional anti-rheumatic drugs alone or in combination [3]. However, the majority of RA patients do not respond to treatment and have acquired drug resistance [4]. Human fibroblast-like synoviocytes (FLSs) are an integral component of the synovial lining [5]; studies have shown that in RA, abnormal hyperplasia of the synovium frequently occurs, forming pannus-like structures, causing tissue destruction and functional impairment [5, 6]. Furthermore, proliferative FLSs in the synovial lining tissue have been recognized as a major pathologic feature of RA [6, 7]. Furthermore, FLSs exhibit a tumor-like phenotype and have been shown to be involved in synovial inflammation and cartilage damage [8], suggesting that targeting the oncogenic properties of FLSs may be a practical approach to RA.

Long non-coding RNAs (lncRNAs) are a class of non-protein-coding transcripts with relatively large size (> 200 nucleotides) [9]. Recently, it has been shown that lncRNAs function as competing endogenous RNAs (ceRNAs), competing with protein-coding mRNAs and binding to miRNAs by sponging miRNAs. LncRNAs have been recognized as essential regulators in a range of biological processes and pathologies, including RA [10]. It has been reported that lncRNAs are differentially expressed in FLSs of RA patients compared to FLSs of healthy individuals [10, 11], suggesting that targeting lncRNAs may be effective. NEAT1) has been reported as an oncogene overexpressed in many malignancies [12]. A recent study reported that elevated NEAT1 expression is associated with inflammatory progression in RA patients via the miR-410-3p/YYY1 axis [13], suggesting that NEAT1 may be a potential biomarker and target for diagnosis and treatment of RA patients.

Cellular metabolism, such as glucose and glutamine metabolism, has been implicated in the activation of FLSs [14, 15], suggesting that targeting metabolic pathways may be a novel approach to treat RA by modulating synovial cell proliferation effectively. However, the exact role and molecular targets of lncRNA NEAT1 in RA pathogenesis remain unclear. In this study, we comprehensively investigated the biological function of NEAT1 in H_2_O_2_-induced FLSs dysfunction of RA. The results showed that NEAT1 was significantly expressed in FLSs-RA compared to normal FLSs. In addition, glutamine metabolism was significantly elevated in RA FLSs. We plan to identify the molecular targets of NEAT1 that regulate glutamine metabolism in FLSs-RA.

## Methods

### Primary culture of FLSs

Human FLSs from normal and rheumatoid arthritis (RA) patients were purchased from Cell Applications (San Diego, CA, USA). Cells were grown in DMEM (ThermoFisher, Carlsbad, CA, USA) containing 10% FBS (Biorad, Hercules, CA, USA), 100 units/ml penicillin, 100 μg/ml streptomycin (ThermoFisher, Carlsbad, CA, USA) at 37 °C in a CO_2_ atmosphere with 5% humidity. The adherent cells were cultured and passaged as before for further use.

### Transfection of siRNA, miRNA and plasmid DNA

FLSs (4 × 10^5^ cells/well) were seeded in 6-well plates and cultured for 24 h to 70% confluency. Transfections of siRNA, miRNA and plasmid DNA into FLSs were performed using the Lipofectamine 2000 transfection reagent (Invitrogen, Carlsbad, CA, USA) according to the manufacturer’s instructions. miRNA or siRNA was transfected at 25 nM for 48 h and plasmid or control vector at 1 µg/well for 48 h. Each experiment was repeated three times independently.

### Computional prediction of ncRNA-miRNA and miRNA-mRNA interactions

The possible interaction between NEAT1-miR-338-3p and miR-338-3p-GLS was analyzed according to the previous description from starBase [16].

### RNA isolation and real-time PCR

Total RNA was extracted from synovial cells using TRIzol reagent (Invitrogen, Carlsbad, CA, USA) according to the manufacturer's instructions; RNA sample quality and quantity were determined using NanoDrop 2000/2000c Spectrophotometers (ThermoFisher, Carlsbad, CA, USA). cDNA synthesis was performed with 1 μg of total RNA using the Revert Aid First Strand cDNA Synthesis kit (Takara, Shiga, Japan) according to the manufacturer's instructions. qRT-PCR reactions were performed using Taqman 2 × Universal PCR Master Mix (ThermoFisher, Carlsbad, CA, USA). qRT-PCR cycling conditions were as follows: 95 °C, 10 min; 95 °C, 30 s, 55 °C, 20 s, 72 °C, 20 s 55 °C, 20 s, 72 °C, 32 cycles of amplification; then 72 °C, 1 min. Results were standardized with GAPDH or U6. PCR primers were synthesized by Sangon Biotech (Shanghai, China). Relative expression levels were calculated by the 2-ΔΔCt method. Experiments were performed in triplicate and repeated three times independently.

### Luciferase assay

Luciferase assays were performed using the dual-luciferase assay system (Promega, Madison, WI, USA) according to the manufacturer's instructions. Wild-type (WT) and mutant (Mut) miR-338-3p binding sites were amplified by PCR and subsequently cloned into the pMiR-report luciferase vector (Promega, Madison, WI, USA). After treatment, FLSs (1 × 10^5^ cells/well) were seeded in 24-well plates and incubated for 24 h. Cells were co-transfected with WT- or Mut- NEAT1 or GLS 3'UTR luciferase reporter vectors with control miRNA or miR-338-3p using Lipofectamine 3000 (Life Technologies, USA). Renilla luciferase was used as an internal control. Experiments were performed in triplicate and repeated three times.

### RNA pull-down assay

For the RNA pull-down assay, FLSs lysates were incubated with biotin-labeled Scramble control, Scramble control, sense, antisense NEAT1 probe synthesized by RiboBio Co. Ltd (Guangzhou, China). The mixture was incubated for 2 h. After incubation of this mixture for 2 h, streptavidin-coupled agarose beads (ThermoFisher, Shanghai, China) were added for 2 h. After washing with PBS, the miR-338-3p from the pulled-down RNA-RNA complexes was measured by qRT-PCR. Experiments were performed in triplicate and repeated three times.

### Measurement of glutamine metabolism

Glutamine metabolism was assessed by measuring glutamine intake and glutaminase activity using the Glutamine and Glutamate Determination Kit (Sigma-Aldrich, Shanghai, China) and the PicoProbeTM Glutaminase (GLS) Activity Assay Kit (BioVision, Milpitas, CA, USA) were evaluated by measuring glutamine intake and glutaminase activity according to the manufacturer's instructions. Results were normalized by the number of cells in each reaction. Experiments were performed in triplicate and repeated three times.

### Clonogenic assay

FLSs were seeded in 6-well plates (5 × 10^3^ cells/well) and cultured in control or hypoglutamine medium for 3 weeks. The culture medium was changed every 2 days. Colonies were fixed and stained with 1.25% crystal violet for 5 min at room temperature; after washing extensively with PBS to remove background dye, colonies were observed by microscopy under a bright field. Each experiment was performed three times independently.

### Cell viability

The viability of FLSs in response to H_2_O_2_ treatment was determined using the 3-(4,5-dimethylthiazol-2-yl)-2,5-diphenyltetrazolium bromide (MTT) assay (Sigma-Aldrich, Shanghai, China) according to the manufacturer's protocol FLSs (1 × 10^3^ cells/ wells) were seeded in 96-well plates and incubated for 24 h. After H_2_O_2_ treatment with 250, 500, or 1000 μM for 48 h, 20 µl of MTT reagent was added to each well and incubated for 4 h at 37 °C. Dimethyl sulfoxide (DMSO) (100 μl) was added to each well and incubated for 1 h. The absorbance was measured at 570 nm using a SpectraMax M5 microplate reader. Experiments were performed in triplicate and repeated three times independently.

### Cell apoptosis assay

The cell death rate of FLSs in response to H_2_O_2_ treatment was determined using the Annexin V/FITC Apoptosis Detection Kit (BD Biosciences, San Jose, CA, USA) according to the manufacturer's protocol. FLAs-RA or control FLSs (4 × 10^5^ cells/well) were plated in 6-well plates for 24 h. After treatment, cells were trypsinized, washed with PBS, and incubated with Annexin V and propidium iodide (PI) for 40 min in the dark. Apoptosis rates were assessed by the FACScan instrument (BD Biosciences, San Jose, CA, USA). Experiments were performed in triplicate and repeated three times.

### Western blotting

FLSs were collected and lysed by RIPA lysis buffer (Beyotime Biotechnology, Nantong, China) with 1 × protease inhibitor cocktail (Sigma-Aldrich, Shanghai, China). Lysates were centrifuged at 12,000 × *g*, 4 °C for 20 min. Protein concentrations were measured by the Bradford assay. Equal amounts (40 μg) of total protein from each group were loaded onto a 10% SDS-PAGE gel and transferred to a nitrocellulose membrane. The membranes were blocked with 5% BSA in TBST buffer for 1 h at room temperature. Membranes were incubated with primary antibody (1:1000) overnight at 4 °C. After thorough washing with TBST, membranes were incubated with secondary antibody (1:3000) for 1 h at room temperature. Protein bands were visualized using the Hyperfilm-ECL kit (GE Healthcare Biosciences). The experiment was repeated three times.

### Statistical analysis

Statistical analysis was performed using GraphPad Prism 7 software (GraphPad. La Jolla, CA, USA). Quantitative data were presented as mean ± standard deviation (SD). For normally distributed variables, differences were analyzed by Student's t-test (between two groups) or one-way analysis of variance (ANOVA) (between three or more groups) followed by Tukey's test. Mann–Whitney's U test was used to determine differences between two data not belonging to a normal distribution. *p* < 0.05 was considered statistically significant.

## Results

### lncRNA NEAT1 and miR-338-3p are inversely expressed in human FLSs-RA

**Fig. 1:**
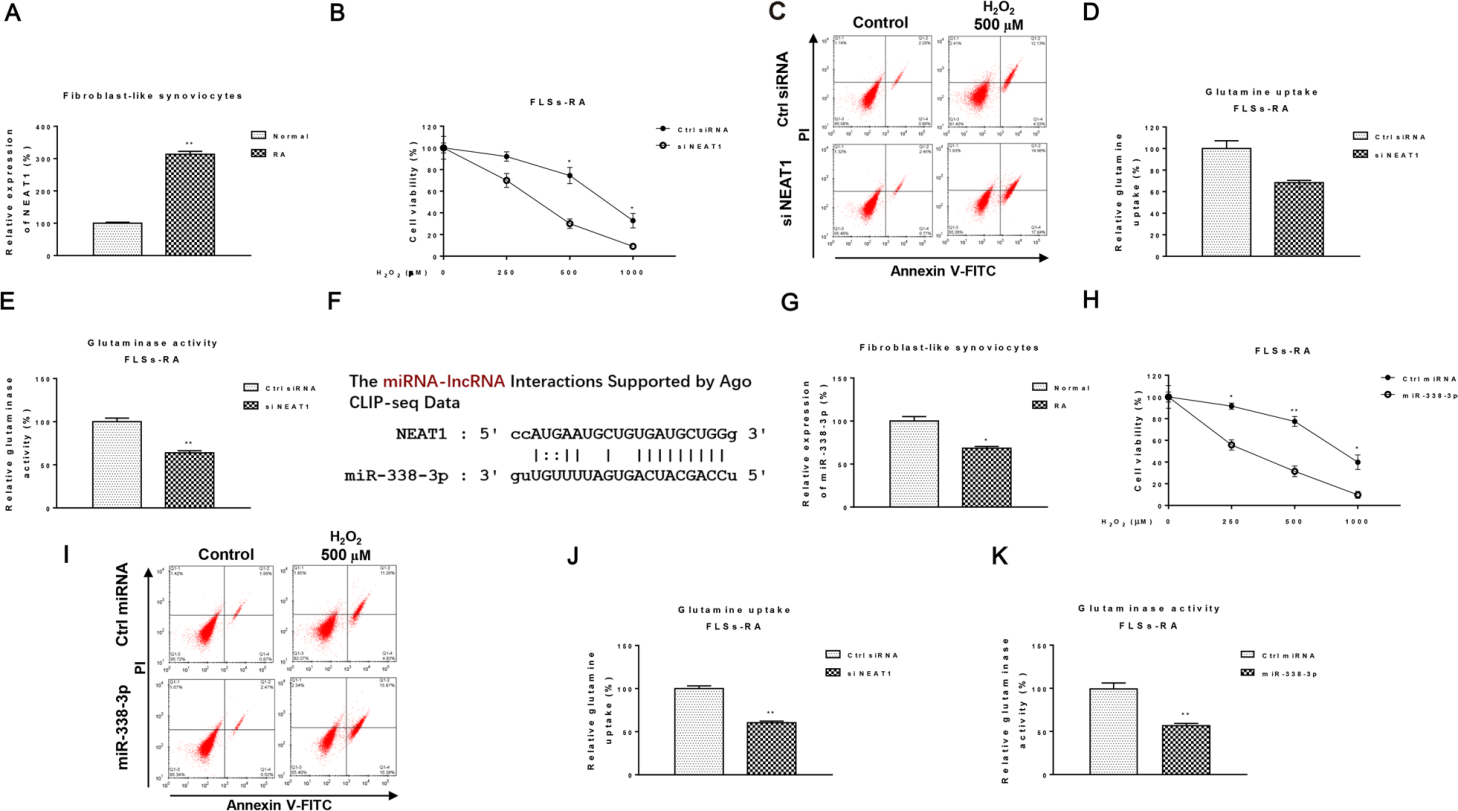
correlation of NEAT1 and miR-338-3p with cellular dysfunction and glutamine metabolism in FLSs-RA. **A** Expression of NEAT1 in FLSs from healthy donors and RA patients. **B** FLSs-RA cells without or with NEAT1 silencing by NEAT1 siRNA transfection were treated with the indicated concentrations of H_2_O_2_ and cell viability was determined by MTT assay, **C** Annexin V assay. **D** Glutamine uptake and **E** glutaminase activity were examined in FLSs-RA cells without and with NEAT1 silencing. **F** Predicted NEAT1-miR-338-3p interaction from StarBase. **G** Expression of miR-338-3p in FLS of healthy donors and RA patients. **H** FLSs-RA cells that did or did not overexpress miR-338-3p were treated with the indicated concentrations of H_2_O_2_, and cell viability was determined by MTT assay and **I** Annexin V assay. **J** Glutamine uptake and **K** glutaminase activity were examined in FLSs-RA cells without or with overexpression of miR-338-3p. Significant differences between the two groups were identified using the Mann–Whitney U test. Values are means (± SD, *n* = 3). *p < 0.05, **p < 0.01

We evaluated the role of lncRNA NEAT1 expression in human fibroblast-like synoviocytes from normal donors and RA patients. Previous studies have shown that FLSs-RA exhibits conventional tumor cell characteristics, such as tumor-like proliferation, migration, invasion, and resistance to cell death [8]. In addition, NEAT1, a lncRNA, is known to be an oncogenic molecule in various cancers [12]. Therefore, we investigated the biological role of NEAT1 in FLSs-RA cells. As expected, NEAT1 expression was significantly elevated in human FLSs-RA compared to normal FLSs (Fig. 1A) To assess the cellular function of NEAT1 in FLSs-RA, we tested apoptosis and nutrient metabolism; FLSs-RA deficient in NEAT1 showed a significant increase in apoptosis induction substance, H_2_O_2_ 500 μM and 1000 µM, for 8 h. The expected results from the cell viability assay and Annexin V apoptosis assay showed that FLSs-RA with low NEAT1 had a significantly increased cell death rate in response to H_2_O_2_ (Fig. 1B, C). Interestingly, glutamine uptake and the rate of glutamine metabolism, as reflected in glutaminase activity, were suppressed in FLSs-RA lacking NEAT1 (Fig. 1D, E). lncRNAs target miRNAs and inhibit their expression [17]. We next searched for potential targets of NEAT1. We then searched for potential targets of NEAT1. Computational prediction by the non-coding RNA online service starBase 2.0 showed that miRNA-338-3p, which has been reported to be negatively correlated with various cancers, contains a binding site for NEAT1 (Fig. 1F). The qRT-PCR results in Fig. 1G showed that miR-338-3p was significantly downregulated in FLSs-RA compared to normal FLSs. Next, we evaluated the role of miR-338-3p in cell death and glutamine metabolism in FLSs-RA. As expected, overexpression of miR-338-3p effectively promoted cell death in FLSs-RA (Fig. 1H, I) and similarly inhibited the rate of glutamine metabolism (Fig. 1J, K). Based on these results, we conclude that NEAT1 positively correlates with FLSs in RA and that miR-38-3p induces dysfunction of FLSs-RA and inhibits it.

### LncRNA NEAT1 sponges miR-338-3p and forms ceRNA networks in FLSs-RA

**Fig. 2:**
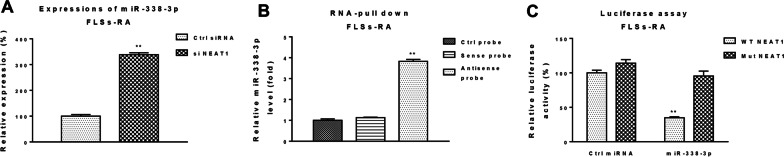
NEAT1 downregulates miR-338-3p by sponging as ceRNA. **A** FLSs-RA was transfected with control siRNA or NEAT1 siRNA and miR-338-3p expression was detected by qRT-PCR. **B** FLSs-RA cell lysates were incubated with biotin-labeled control, sense or antisense NEAT1 probes for pull-down assays. miR-338-3p enrichment was assessed by qRT-PCR. **C** Luciferase reporter assays were performed by transfection of FLSs-RA with luciferase vectors containing WT- or Mut-NEAT1 and control miRNA or miR-338-3p. Luciferase activity was examined; significant differences between the two groups were identified using the Mann–Whitney U test. Values are means (± SD, *n* = 3). *p < 0.05, **p < 0.01

To assess the regulatory mechanisms underlying NEAT1-mediated glutamine metabolism and FLS dysfunction in RA patients. We tested the predicted NEAT1-miR-338-3p association; FLSs-RA with NEAT1 silencing showed significant upregulation of miR-338-3p (Fig. 2A), indicating that NEAT1 blocks miR-338-3p expression via sponging. The results of this study showed that NEAT1 blocked miR-338-3p expression via sponging. In addition, RNA pull-down assays were performed by incubating biotin-labeled control, sense or antisense probes of NEAT1 with cell lysates of FLSs-RA. The results (Fig. 2B) showed that miR-338-3p was enriched only in the antisense probe-pulldown RNA complex. To test whether NEAT1 directly binds to miR-338-3p, FLSs from RA patients were incubated with wild-type NEAT1 (WT-NEAT1) or binding site mutant NEAT1 (Mut-NEAT1) co-transfected with a luciferase vector containing control miRNA or miR-338-3p. Luciferase assays consistently showed that the luciferase activity of wild-type and miR-338-3p co-transfected FLSs was clearly inhibited compared to that of co-transfection of Mut-NEAT1 and miRNA-338-3p (Fig. 2C). These results indicate that NEAT1 sponges miR-338-3p in FLSs-RA and downregulates it by forming a ceRNA network.

### Glutamine metabolism is elevated in FLSs derived from RA patients

**Fig. 3:**
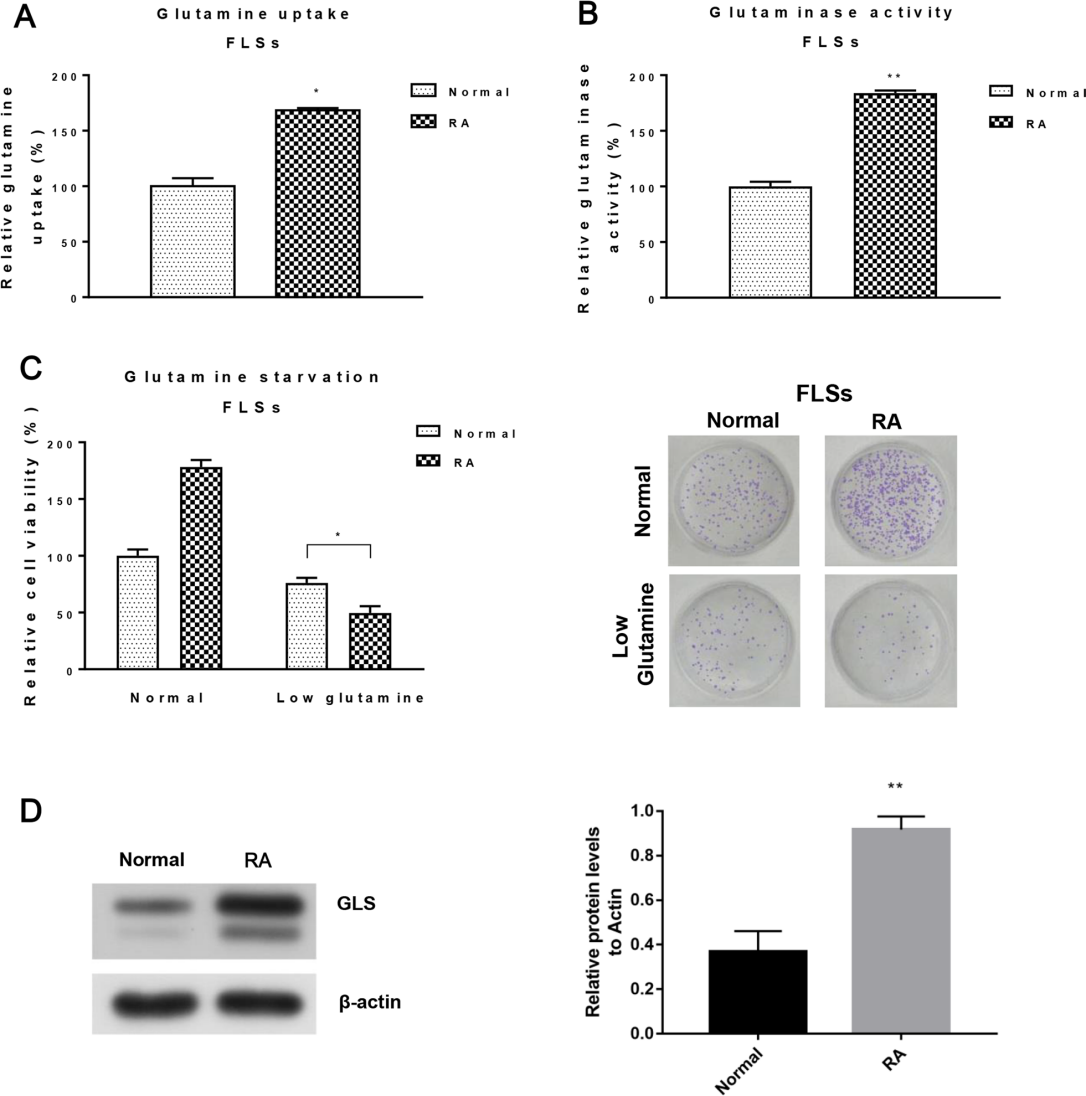
The rate of glutamine metabolism is increased in FLSs-RA. **A** Glutamine uptake and **B** glutaminase activity were examined in FLSs from normal subjects and RA patients. **C** FLS of normal and RA patients were cultured under normal or low glutamine conditions. Cell viability was examined by clonogenic and MTT assays. **D** Protein expression of GLS in FLS of normal and RA patients was detected by Western blotting. β-actin served as an internal control. The significant differences between the two groups were identified using the Mann–Whitney U test. Values are means (± SD, *n* = 3). *p < 0.05, **p < 0.01

We have shown that NEAT1 enhances glutamine metabolism, and miR-338-3p inhibits it in FLSs derived from RA patients. To investigate the effect of glutamine metabolism in FLSs-RA, we examined glutamine uptake and glutaminase in healthy and RA patient FLSs. Compared to healthy FLSs, glutamine uptake and glutaminase activity were significantly increased in FLSs-RA (Fig. 3A, B). Furthermore, under low glutamine conditions, FLSs-RA significantly suppressed cell viability compared to that of healthy FLSs (Fig. 3C). This suggests that FLSs-RA has glutamatergic properties. In addition, protein expression of glutaminase (*), which catalyzes the conversion of glutamine to glutamate, the rate-limiting step in glutamine metabolism, was clearly elevated in FLSs-RA (Fig. 3D). These results suggest that targeting the abnormal glutamine metabolism in FLSs-RA could inhibit FLSs proliferation and potentially treat RA.

### miR-338-3p targets GLSs and inhibits glutamine metabolism in FLSs-RA

**Fig. 4:**
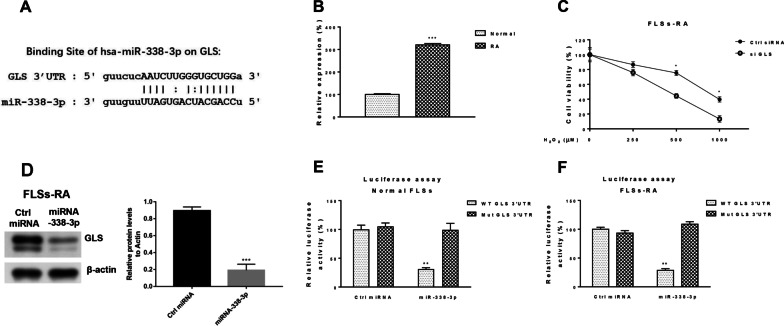
miR-338-3p directly targets the 3' UTR of the GLS of FLSs. **A** miR-338-3p binding sites on the 3' UTR of GLSs were predicted from StarBase. **B** The expression of GLS mRNA in normal FLSs and FLSs-RA was detected by qRT-PCR. **C** FLSs-RA cells were transfected with control siRNA or GLS siRNA for 48 h, and cells were treated with the indicated concentration of H_2_O_2_. Cell viability was examined by MTT assay. **D** FLSs-RA cells were transfected with control miRNA or miR-338-3p, and protein expression of GLS was detected by Western blot. **E** WT- or Mut-3'UTR of GLS was transfected into normal FLSs and **F** FLSs-RA with control miRNA or miR-338-3p, and a luciferase reporter assay was performed. Luciferase activity was examined; significant differences between the two groups were identified using the Mann–Whitney U test. Values are means (± SD, *n* = 3). *p < 0.05, **p < 0.01

Many studies have shown that microRNAs repress the expression of target mRNAs by binding directly to the 3'UTR of mRNAs [17]. miR-338-3p target candidates were searched in starBase. We found a binding site for miR-338-3p in the 3'UTR of GLS (Fig. 4A). miR-338-3p was significantly upregulated in FLSs-RA cells compared to normal synovial cells (Fig. 4B). siRNA inhibition of GLS expression promoted H_2_O_2_-induced cell death (Fig. 4C), suggesting that GLS is positively correlated with the progression of FLSs in RA patients. Consequently, overexpression of miR-338-3p markedly blocked the expression of GLS proteins in FLSs-RA cells (Fig. 4D). To test whether miR-338-3p directly targets the 3'UTR of GLSs, we tested the expression of GLSs in the wild-type or binding site mutant 3'UTR containing Luciferase reporter assays were performed by co-transfection of luciferase vectors and control miRNAs or miR-338-3p into normal FLSs and FLSs-RA. Luciferase activity in cells co-transfected with the 3′UTR of wild-type GLSs and miR-338-3p was significantly inhibited compared to controls (Fig. 4E, F). These results confirm that miR-338-3p directly targets the 3'UTR of GLSs in FLSs.

### Restoration of GLS restored miR-338-3p-mediated glutamine metabolism and apoptosis in FLSs-RA

**Fig. 5:**
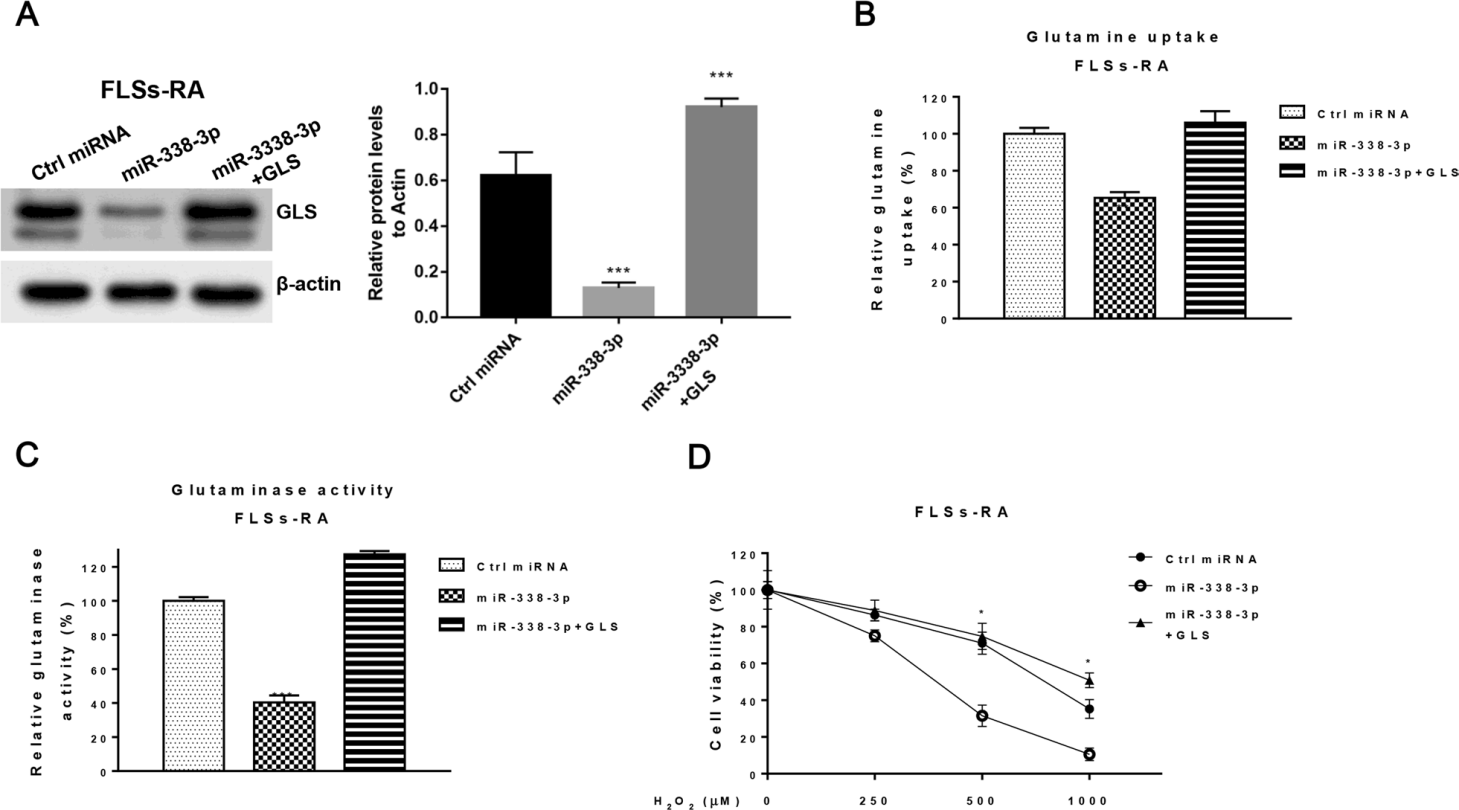
Restoration of GLS restored miR-338-3p-mediated glutamine metabolism and cellular dysfunction in FLSs-RA. **A** FLSs-RA were transfected with control, miR-338-3p alone, or GLS plus, and GLS expression was examined by Western blot. **B** Glutamine uptake and **C** glutaminase activity were detected from the above-transfected cells. **D** FLSs-RA cells transfected with control, miR-338-3p alone, or plus GLS were treated with H_2_O_2_ at the indicated concentrations for 8 h, and cell viability was determined by MTT assay. Significant differences between the two groups were identified using the Mann–Whitney U test. Values are means (± SD, *n* = 3). *p < 0.05, **p < 0.01, ***p < 0.001

To assess whether miR-338-3p-mediated FLSs-RA apoptosis and glutamine metabolism are targeted by GLS, rescue experiments were performed by transfecting FLSs-RA with control miRNA, miR-338-3p alone or GLS overexpression vector. When GLS was exogenously transfected into FLSs-RA cells overexpressing miR-338-3p, GLS protein expression was successfully rescued (Fig. 5A). As a result, restoration of GLS effectively restored glutamine uptake and glutaminase activity (Fig. 5B, C). As expected, GLS-mediated the restoration of glutamine metabolism rendered FLSs-RA cells resistant to apoptosis-inducing agents based on cell viability measurements (Fig. 5D). In summary, the rescue experiments consistently showed that miR-338-3p suppresses glutamine metabolism by targeting GLS and induces dysfunction of FLSs-RA.

### LncRNA NEAT1 suppresses miR-338-3p-GLS-mediated FLSs-RA dysfunction

**Fig. 6:**
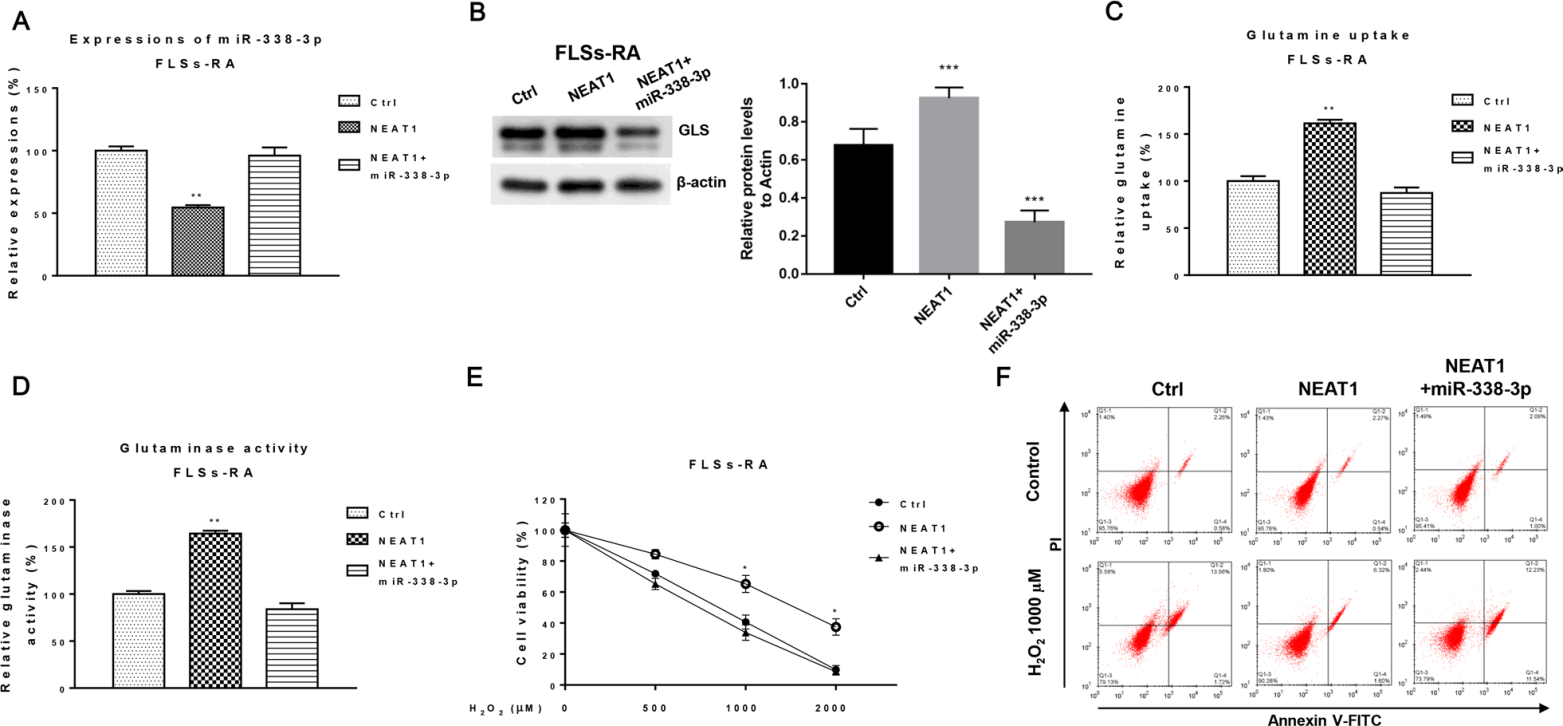
Role of the NEAT1-miR-338-3p-GLS axis in FLSs-RA. **A** FLSs-RA was transfected with control, NEAT1 alone, or in combination with miR-338-3p, **B** protein expression of GLS was examined by qRT-PCR and Western blot, respectively. **C** Glutamine uptake assay and **D** glutaminase activity were measured from the above-transfected cells. **E** FLSs-RA were transfected with control, NEAT1 alone or in combination with miR-338-3p, cells were treated with H_2_O_2_ at the indicated concentrations for 48 h and cell viability was determined by MTT assay and **F** Annexin V apoptosis assay. Significant differences between the two groups were identified using the Mann–Whitney U test. Values are means (± SD, *n* = 3). *p < 0.05, **p < 0.01

Finally, we evaluated whether NEAT1 modulates H_2_O_2_-induced FLSs-RA apoptosis by targeting the miR-338-3p-GLS-glutamine metabolic axis. FLSs-RA cells were co-transfected with negative control, NEAT1 overexpression vector alone, or miR-338-3p were used. As expected, NEAT1 overexpression suppressed miR-338-3p and was further rescued by miR-338-3p overexpression (Fig. 6A). Furthermore, Western blot results showed that NEAT1 overexpression significantly upregulated GLS expression, which was overcome by transfection of miR-338-3p (Fig. 6B). Furthermore, restoration of miR-338-3p in NEAT1-overexpressing FLSs-RA effectively repressed glutamine uptake and GLS activity (Fig. 6C, D) and rescued H_2_O_2_-induced cell death in FLSs-RA (Fig. 6E, F). These data consistently validated that NEAT1 impairs H_2_O_2_-induced FLSs-RA cell dysfunction by modulating the miR-338-3p-GLS pathway, presenting NEAT1 as a promising biomarker and molecular target for the diagnosis and treatment of rheumatoid arthritis.

## Discussion

Rheumatoid arthritis (RA) is a systemic chronic autoimmune disease [1, 2]. Currently, the majority of RA patients do not respond to treatment and have developed drug resistance [3, 4]. Studies have shown that fibroblast-like synoviocytes (FLSs), an important component of synovial tissue, play an essential role in the progression of the immune response in RA patients [5, 6]. In RA, FLSs are known to hyperproliferate and form pannus-like structures, which increase their migration into articular cartilage and secrete diverse inflammatory cytokines, further exacerbating the pathogenesis of RA [7]. Therefore, it is an important task to understand the underlying molecular mechanisms of FLSs dysregulation in RA. In this study, we aimed to determine the biological roles and molecular targets of lncRNA NEAT1 in cellular metabolism and dysfunction of FLSs. The results showed that the expression of NEAT1 was significantly upregulated in FLSs of RA patients compared to normal FLSs, suggesting that NEAT1 may be a diagnostic biomarker and therapeutic target for RA.

A number of studies have demonstrated that lncRNAs play essential roles in the etiology of diseases, including RA [9, 18]. Recently, expression profiles of lncRNAs by microarray analysis were reported in normal and RA patient FLSs [11]. NEAT1 has been reported to positively correlate with various cancers. However, the role of NEAT1 in FLSs-RA and the downstream targets of NEAT1 are unknown. We found that NEAT1 promotes intracellular glutamine metabolism and decreases apoptosis in FLSs-RA, consistent with the role of NEAT1 in cancer. LncRNAs sponge target miRNAs and downregulate their expression to form ceRNA networks and de-repress miRNA targets [17]. Bioinformatics analysis predicted that miR-338-3p, which is reported to be frequently downregulated in cancer, is a target of NEAT1 [19]. As expected, miR-338 was clearly downregulated in FLSs-RA. miR-338 overexpression effectively suppressed glutamine metabolism and promoted FLSs-RA dysfunction. Luciferase and RNA pull-down assays confirmed that miR-338-3p was sponged by NEAT1 in FLSs-RA, suggesting that NEAT1-mediated cellular metabolism and dysfunction of FLSs are mediated through miR-338-3p.

Numerous studies have shown that FLSs in RA patients exhibit aggressive cancer-like features, including hyperproliferation, increased migratory and invasive potential, elevated cellular metabolism, and resistance to apoptosis. Synovitis is a typical pathological feature of RA, while these inflammatory microenvironments induce metabolic changes in most nutrients, including glucose, glutamine, glutamate, lactate, and oxygen [14]. The most abundant free amino acid in the human body is glutamine, which accounts for approximately 50% of all free amino acids in plasma [20]. Glutamine is hydrolyzed to glutamate and ammonium ions (NH4) by glutaminase (GLS) and synthesized by glutamine synthase (GS) with the consumption of 1 ATP. Significant changes in the activity or expression of GLS or GS can affect glutamine production and cell consumption may have an impact. The present results are consistent with a previous study [15] that showed increased GLS expression and glutamine consumption in FLSs-RA. In addition, cell viability in FLSs-RA was significantly reduced compared to normal FLSs under glutamine deprivation, suggesting that FLSs-RA exhibits features of "glutamine intoxication," in other words, that glutamine degradation is important in the pathogenesis of RA synovitis GLSs include kidney-type glutaminase (GLS1) and liver-type glutaminase (GLS2) isoforms have been identified, which differ in kinetic properties, protein structure, and organization [21]. In cancer cells, the two isozymes serve contrasting functions in tumorigenesis; GLS1 is associated with tumor growth and malignant transformation modified by the cancer-associated protein c-Myc, whereas GLS2's ability is tumor suppression, which is manipulated by p53 [22–24]. Several previous studies have found that Myc expression is elevated in fibroblast-like synoviocytes of RA patients, raising the possibility that GLS1 expression is positively regulated by MYC in FLSs-RA [15, 25]. Many investigators have noted elevated inflammatory cytokines and chemokines in synovial fluid, synovium, cytoplasm, and culture medium in FLSs such as arthritic synovitis [26, 27]. Takahashi et al. found that TNF-α, IL-1β, and IL-6 did not immediately promote GLS1 production in FLS, and only IL-17 and PDGF modulated GLS1 expression [15].

Subsequently, we identified GLS, a key enzyme in glutamine metabolism, as a direct target of miR-338-3p in FLSs-RA. miR-338-3p post-transcriptionally regulates glutamine metabolism and apoptosis in FLSs-RA by targeting GLS messenger RNA rescue experiments verified that miR-338-3p regulates glutamine metabolism and apoptosis in FLSs-RA by targeting and post-transcriptionally modulating GLS messenger RNA. Furthermore, overexpression of NEAT1 in FLSs-RA effectively restored the ability of miR-338-3p to repress GLS expression. In general, we found that lncRNA NEAT1 may result in the expression of GLSs that promotes glutamine metabolism and an anti-apoptotic phenotype through the regulation of miR-338-3p. This study suggests that inhibition of the lncRNA NEAT1 pathway may be a proactive approach to RA.

However, this study has several limitations that need to be clarified. This study did not include an animal model of RA to validate cell-based conclusions; the main features of RA are synovitis and the production of inflammatory mediators, but this study only examined GLS expression regulated by NEAT1. Further studies on the relationship between inflammatory mediators and free amino acids, especially glutamine, will be needed over time. Thousands of microRNAs are involved in sponge binding, suggesting that target genes belong to different signaling pathways or are affected by intermolecular crosstalk.

## Conclusions

In summary, we have identified the critical role and molecular mechanisms of NEAT1-mediated glutamine metabolism and FLSs-RA dysfunction through the regulation of the miR-338-3p-GLS pathway, and targeting glutamine metabolism by non-coding RNAs to inhibit FLSs overgrowth for the treatment of RA. Therefore, targeting glutamine metabolism by non-coding RNAs may be a practical therapeutic approach.

## Data Availability

The datasets used or analyzed in this study are available from the corresponding author upon reasonable request.
